# Characteristics and Outcomes of Patients Deferred for Transcatheter Aortic Valve Replacement Because of COVID-19

**DOI:** 10.1001/jamanetworkopen.2020.19801

**Published:** 2020-09-30

**Authors:** Richard Ro, Sahil Khera, Gilbert H. L. Tang, Parasuram Krishnamoorthy, Samin K. Sharma, Annapoorna Kini, Stamatios Lerakis

**Affiliations:** 1 Division of Cardiology, Icahn School of Medicine at Mount Sinai, Mount Sinai Hospital, New York, New York; 2 Department of Cardiovascular Surgery, Icahn School of Medicine at Mount Sinai, Mount Sinai Hospital, New York, New York

## Abstract

This cohort study examines the characteristics and outcomes of patients for whom transcatheter aortic valve replacement (TAVR) was deferred because of the coronavirus disease 2019 (COVID-19) pandemic.

## Introduction

Coronavirus disease 2019 (COVID-19) is a global pandemic that has led to diversion of resources to the front lines and postponement of elective procedures. Patients with structural heart disease are a high-risk cohort because of their age and comorbidities. Management of their underlying condition has sometimes been delayed as a result of efforts to avoid community and health care setting exposure to COVID-19. An executive order was enacted by the New York State government on March 22, 2020, leading to cancellation of elective procedures. We describe here the outcomes of patients with symptomatic, severe aortic stenosis (AS) from our structural heart disease program during the COVID-19 pandemic.

## Methods

This was a single-center cohort study of 77 patients with severe AS undergoing evaluation for transcatheter aortic valve replacement (TAVR) at a tertiary care hospital before the COVID-19 pandemic. This study was conducted under an institutional review board for the Structural Heart Program of Mount Sinai Hospital. The study posed minimal risk to patients, and the collected data were deidentified; thus, the need for informed consent was waived. This study follows the Strengthening the Reporting of Observational Studies in Epidemiology (STROBE) reporting guideline.

A cardiac event was defined as the need for urgent TAVR or death. Patients were followed up for 3 months. All data were compiled by weekly telephone calls and medical record review. Continuous variables are expressed as mean (SD), and categorical variables are expressed as percentages. The *t* test was used for continuous variables. For nonnormally distributed data, the Kruskal-Wallis test was used. The χ^2^ test was used for categorical variables. A 2-tailed α of .05 was considered the threshold for statistical significance for all tests. Statistical analysis was performed using Stata MP statistical software version 14.0 (StataCorp). Data analysis was performed from March to June 2020.

## Results

Of the 77 patients (mean [SD] age, 80 [8] years; 49 men [64.0%]), 55 (71.4%) had originally been scheduled for TAVR. Twenty-two patients (28.6%) had diagnostic testing or heart team appointments canceled because of COVID-19. Patient characteristics are shown (Table 1). During the initial 1-month period between March 23 and April 21, 2020, 8 of 77 (10%) experienced a cardiac event. Six patients underwent TAVR urgently for accelerating symptoms of dyspnea, angina at rest, heart failure, or syncope. Two patients died of severe AS. Those with a cardiac event, compared with those with no event, had a significantly lower left ventricular ejection fraction (mean [SD], 45% [16%] vs 56% [14%]; difference, 11%; 95% CI, 0.3%-21%; *P* = .04), a higher incidence of obstructive coronary artery disease (7 patients [87.5%] vs 35 patients [50.7%]; χ^2^_1_ = 3.9; *P* = .048), and higher incidence of New York Heart Association class III (7 patients [87.5%] vs 26 patients [37.7%]) and class IV (1 patient [12.5%] vs 1 patient [1.4%]) symptoms (χ^2^_4_ = 12.4; *P* = .02) (Table 1).

**Table 1.** Patient Characteristics for the Total Cohort and According to Cardiac Events Between March 23 and April 21, 2020

Characteristic	Patients, No. (%)			*P* value
	Total study cohort (N = 77)	TAVR or death (n = 8)	No TAVR or death (n = 69)	
Age, mean (SD), y	80 (8)	82 (8)	79 (8)	.90
Male	49 (64.0)	4 (50.0)	45 (65.2)	.40
Body mass index, mean (SD)^a^	27.2 (5)	27.4 (5)	27.2 (4)	.90
Hypertension	73 (94.0)	8 (100.0)	65 (94.2)	.40
Diabetes	34 (44.2)	3 (37.5)	31 (44.9)	.70
Chronic kidney disease	37 (48.1)	3 (37.5)	34 (49.2)	.50
End-stage kidney disease	8 (10.1)	1 (12.5)	7 (10.1)	.80
Left ventricular ejection fraction, mean (SD), %	55 (14)	45 (16)	56 (14)	.04
Cerebrovascular accident	9 (11.4)	2 (25.0)	7 (10.1)	.20
Transient ischemic attack	2 (2.5)	0	2 (2.9)	.60
Prior permanent pacemaker	7 (8.9)	1 (12.5)	6 (8.7)	.70
Coronary artery bypass grafting	11 (14.3)	2 (25.0)	9 (13.0)	.40
Coronary artery disease	42 (54.5)	7 (87.5)	35 (50.7)	.048
Atrial fibrillation	24 (31.2)	2 (25.0)	22 (31.9)	.70
Congestive heart failure	29 (37.7)	5 (62.5)	24 (34.8)	.10
Aortic valve gradient, mean (SD), mm Hg	34 (13)	36 (9)	34 (14)	.80
Aortic valve area, mean (SD), cm^2^	0.8 (0.2)	0.7 (0.2)	0.8 (0.2)	.30
New York Heart Association symptom class				
I	8 (10.4)	0	8 (11.6)	.02
II	31 (40.3)	0	31 (44.9)	
III	33 (42.9)	7 (87.5)	26 (37.7)	
IV	2 (2.6)	1 (12.5)	1 (1.4)	

Abbreviation: TAVR, transcatheter aortic valve replacement.

^a^ Body mass index is calculated as weight in kilograms divided by height in meters squared.

We next performed an analysis where patients were followed up beyond the 1-month period, to June 6, 2020, when elective procedures at our hospital were permitted to resume. Between March 23 and June 6, 2020, 27 of 77 patients (35%) experienced a cardiac event, with 24 requiring urgent TAVR for accelerated symptoms and 3 dying. During this period, those who experienced a cardiac event, compared with those who did not, were more likely to have a history of cerebrovascular accident (6 patients [22.2%] vs 3 patients [6.0%]; χ^2^_1_ = 4.5; *P* = .03) and New York Heart Association class III (22 patients [81.5%] vs 26 patients [52.0%]) and class IV (2 patients [7.4%] vs 1 patient [2.0%]) symptoms (χ^2^_4_ = 10.6; *P* = .03). Patients who experienced a cardiac event had slightly lower left ventricular ejection fraction compared with those who did not but the difference was not significant (mean [SD], 52% [15%] vs 57% [13%]; difference, 5%; 95% CI, 0%-12%; *P* = .10) (Table 2). In the 3 months before March 2020, no patients died of AS while awaiting TAVR.

**Table 2.** Patient Characteristics According to Cardiac Events Between March 23 and June 6, 2020

Characteristic	Patients, No. (%)		*P* value
	TAVR or death (n = 27)	No TAVR (n = 50)	
Age, mean (SD)	80 (8)	80 (8)	.90
Male	15 (55.6)	33 (66.0)	.40
Body mass index, mean (SD)^a^	28 (5)	27 (4)	.60
Hypertension	26 (96.3)	47 (94.0)	.70
Diabetes	11 (40.7)	24 (48.0)	.50
Chronic kidney disease	12 (44.4)	26 (52.0)	.60
End-stage kidney disease	3 (11.1)	5 (7.2)	.80
Left ventricular ejection fraction, mean (SD), %	52 (15)	57 (13)	.10
Cerebrovascular accident	6 (22.2)	3 (6.0)	.03
Prior permanent pacemaker	4 (14.8)	3 (6.0)	.20
Coronary artery bypass grafting	4 (14.8)	8 (16.0)	.90
Coronary artery disease	16 (59.2)	25 (50.0)	.40
Atrial fibrillation	7 (25.9)	18 (36.0)	.40
Congestive heart failure	13 (48.1)	15 (30.0)	.10
Aortic valve gradient, mean (SD), mm Hg	36 (15)	34 (12)	.60
Aortic valve area, cm^2^	0.8 (0.2)	0.8 (0.2)	.60
New York Heart Association class			
I	0	4 (8.0)	.03
II	3 (11.1)	16 (32.0)	
III	22 (81.5)	26 (52.0)	
IV	2 (7.4)	1 (2.0)	

Abbreviation: TAVR, transcatheter aortic valve replacement.

^a^ Body mass index is calculated as weight in kilograms divided by height in meters squared.

## Discussion

During the COVID-19 pandemic, vigilance is needed for patients with AS awaiting TAVR, because 10% of our patients experienced a cardiac event during the first month, and 35% did so over the course of the next 3 months. We must be judicious when deciding to intervene, because there are additional risks to consider for both the patient and the heart team.^1,2^ In addition, it is necessary to resume required interventions as we pass the initial peak of COVID-19 hospitalizations and health care resources become available. The study was limited by being a single-center study with a limited sample size. Patients with advanced symptoms, lower left ventricular ejection fraction, obstructive coronary artery disease, and cerebrovascular accident history represent a high-risk population with AS, and the heart team should consider these factors for earlier access to TAVR during the COVID-19 pandemic.
